# Medical Imaging Lesion Detection Based on Unified Gravitational Fuzzy Clustering

**DOI:** 10.1155/2017/8536206

**Published:** 2017-10-12

**Authors:** Jean Marie Vianney Kinani, Alberto Jorge Rosales Silva, Francisco Gallegos Funes, Dante Mújica Vargas, Eduardo Ramos Díaz, Alfonso Arellano

**Affiliations:** ^1^Instituto Tecnológico Superior de Huichapan, Domicilio Conocido S/N, Col. El Saucillo, 42411 Huichapan, HGO, Mexico; ^2^Instituto Politécnico Nacional de México, Avenida IPN s/n, Edificio Z, Acceso 3, 3er piso, SEPI-Electrónica, Col. Lindavista, 07738 Ciudad de México, Mexico; ^3^Centro Nacional de Investigación y Desarrollo Tecnológico, Interior Internado Palmira S/N, Palmira, 62490 Cuernavaca, MOR, Mexico; ^4^Universidad Autónoma de la Ciudad de México, Calle Prolongación San Isidro 151, Iztapalapa, San Lorenzo Tezonco, 09790 Ciudad de México, Mexico; ^5^Instituto Nacional de Neurología y Neurocirugía, Av. Insurgentes Sur 3877, Col. La Farma, 14269 Ciudad de México, Mexico

## Abstract

We develop a swift, robust, and practical tool for detecting brain lesions with minimal user intervention to assist clinicians and researchers in the diagnosis process, radiosurgery planning, and assessment of the patient's response to the therapy. We propose a unified gravitational fuzzy clustering-based segmentation algorithm, which integrates the Newtonian concept of gravity into fuzzy clustering. We first perform fuzzy rule-based image enhancement on our database which is comprised of T1/T2 weighted magnetic resonance (MR) and fluid-attenuated inversion recovery (FLAIR) images to facilitate a smoother segmentation. The scalar output obtained is fed into a gravitational fuzzy clustering algorithm, which separates healthy structures from the unhealthy. Finally, the lesion contour is automatically outlined through the initialization-free level set evolution method. An advantage of this lesion detection algorithm is its precision and its simultaneous use of features computed from the intensity properties of the MR scan in a cascading pattern, which makes the computation fast, robust, and self-contained. Furthermore, we validate our algorithm with large-scale experiments using clinical and synthetic brain lesion datasets. As a result, an 84%–93% overlap performance is obtained, with an emphasis on robustness with respect to different and heterogeneous types of lesion and a swift computation time.

## 1. Introduction

In broad terms, “brain lesion” can be defined as an abnormal damage or change in the brain tissue; this can be caused by injury, infection, exposure to certain chemicals, problems with the immune system, and many other factors. Due to their location, at the center of thought, physical function, and emotion, brain lesions are difficult to diagnose and treat. However, thanks to recent advances in magnetic resonance imaging and computing, brain lesion diagnosis has made a great leap. The algorithm presented in this paper could make a huge impact in both diagnosing and monitoring processes. It can detect damaged or unhealthy regions on MRI scans and delineates them with high precision. This facilitates the decision making and the planning of surgical removal of the lesion (if necessary and possible). It also allows one to apply spatially localized radiotherapy, for example, Cyberknife and iMRT [1–3], which in current clinical practice is usually done manually on both contrast-enhanced T1-weighted or FLAIR images. Most of the medical imaging modalities give out images of gray scale intensities, including MRIs. These images are subject to noise, artifacts, and poor resolution and contrast due to instrument and reconstruction algorithm limitations or even patient movement. Consequently, auto-detection becomes so challenging that the algorithm advantages and disadvantages may vary depending on the properties of the image under examination. Therefore, due to the image deterioration factors mentioned, it is hard to develop a standard approach capable of working with all types of MR images [4]. As a result, trade-offs have always been present in computer-aided diagnosis systems. Yet, comparing our unified fuzzy-hard clustering-based system against classical approaches based on methods like classifier, region growing, neural networks, deformable models, and so forth, a big advantage of our approach is recognized especially when dealing with the deteriorating factors mentioned [5]. In Section 2, we present our framework for lesion detection. Starting with a brief background of fuzzy sets, we will show how this theory can be exploited to enhance the inhomogeneous MR images by adapting appropriate fuzzy rules [6]. Next, we will give an outline of the segmentation method used, which is based on a novel gravitational fuzzy clustering concept and level set evolution that defines the final location, shape, and size of the lesion. Experiments and evaluation studies that were carried out on both synthetic and expert-segmented data sets are presented in Section 3. We will then finish the paper with a discussion and a conclusion in Section 4.

## 2. Fuzzy and Gravitational Methods Proposed

### 2.1. The Proposed Fuzzy Set-Based MR Image Enhancement

Accurate diagnosis of brain lesions depends upon the quality of the MR scan; in particular, on the visibility of small, low-contrast objects within the brain image. Unfortunately, the contrast between these objects is often so low that the detection of some abnormalities becomes difficult, especially when dealing with dense tissues. To deal with this issue, contrast enhancement is normally carried out on original images before the detection process can take place.

**Figure 1:**
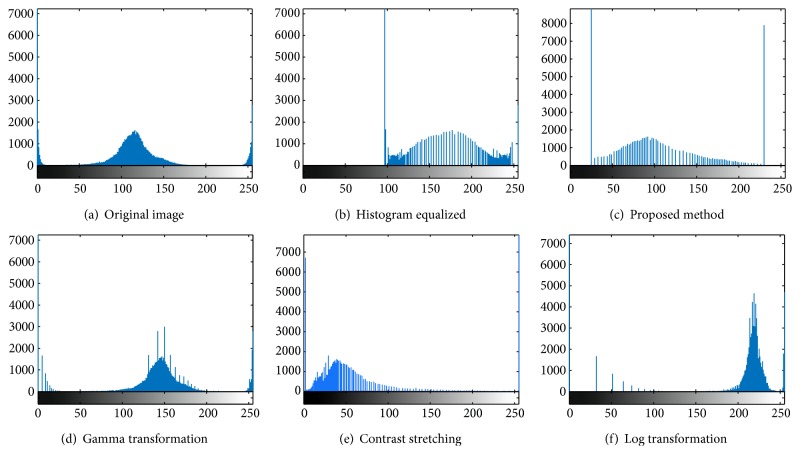
Corresponding histograms.

A detailed investigation on image enhancement carried out by Bankman [7], González and Woods [8], Shih [9], and Russ [10] shows that classical methods like negative transformation, Log, Gamma, contrast stretching, or histogram-based transformations work effectively in enhancing ordinary images; however, when applied to MR images, they bring about tradeoffs between the enhancement and image detail preservation due to the loss of some basic characteristics in the original image histogram, as it is pronounced in Figure 1.

After an intensive study of MR image enhancement techniques, we came up with a much better method based on adapting fuzzy rules. This technique did correct the abovementioned drawback; it has achieved this by enhancing the contrast of features of interest and improving the visibility of diagnostic details without creating artifacts or losing image details as a whole. To get a better understanding of this technique, we have to go back to the fuzzy set theory.

Normally, in set theory, we are used to the so called crisp sets whose membership can only be true or false in the traditional sense of bivalued Boolean logic. This classical set theory has limited use in practical applications due to its lack of flexibility [11]. Thus, Professor Zadeh proposed a more successful theory of fuzzy sets that introduced the idea of partial memberships described by membership functions [11]. Suppose *Z* is a set of elements, and each element is denoted by *z*, that is, *z* ∈ *Z*. A fuzzy subset *A* in *Z* is characterized by a membership function, *μ*_*A*_(*z*), so

(1) $$\begin{array}{c} A=\left\{z,\mu_{A}\left(z\right)\left|z\in Z\right.\right\}. \end{array}$$

When the variables are continuous, *A* in this equation can have an infinite number of elements. When the values of *z* are discrete, the elements of *A* can be shown explicitly. The formalization of the problem into fuzzy rules consists in finding a way to increase the contrast of certain tissues in the brain, while leaving other tissues quasi-untouched to accentuate the difference between these tissues, so the following fuzzy rule is proposed:

(2) $$\begin{array}{c} \begin{array}{l} Fuzzy\;Rule\;1\text{: }IF\;a\;pixel\;is\;dark,THEN\;make\;it\;darker;OR,\\ Fuzzy\;Rule\;2\text{: }IF\;a\;pixel\;is\;gray,THEN\;make\;it\;gray;OR,\\ Fuzzy\;Rule\;3\text{: }IF\;a\;pixel\;is\;bright,THEN\;make\;it\;brighter. \end{array} \end{array}$$

**Figure 2:**
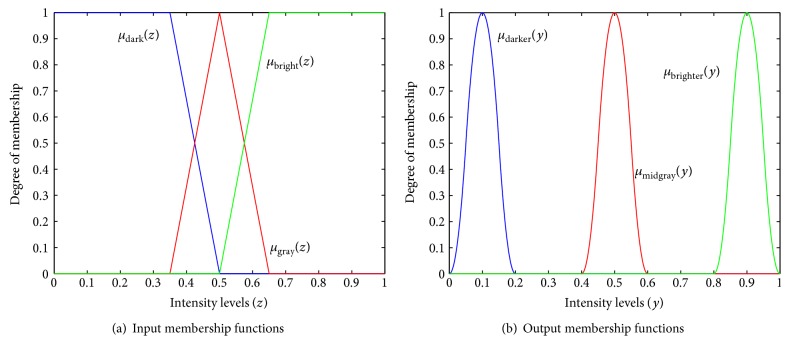
Membership functions.

The first antecedent of the fuzzy rule will seek to relate the fuzzy set dark to the set darker (the two sets are represented in blue in Figure 2()), and the consequence is achieved using a fuzzy *AND* operation, implemented through a min operation [11], as shown in

(3) $$\begin{array}{c} \mu_{1}\left(z,y\right)=\min\left\{\mu_{dark}\left(z\right),\mu_{darker}\left(y\right)\right\}, \end{array}$$

where *z* and *y* are scalar values representing the intensity levels of the pixels in the input and output fuzzy sets, respectively. *z*_0_ denotes a specific intensity level in the interval near to the visible black color spectrum. The degree of membership of the dark set component in response to this input is a scalar value *μ*_dark_(*z*_0_). We find the output corresponding to the first part of the fuzzy rule, and this specific input, by performing the *AND* operation between *μ*_dark_(*z*_0_) and the general result *μ*_1_(*z*, *y*), evaluated also at *z*_0_, so therefore,

(4) $$\begin{array}{c} Q_{1}\left(y\right)=\min\left\{\mu_{dark}\left(z_{0}\right),\mu_{1}\left(z_{0},y\right)\right\}, \end{array}$$

where *Q*_1_(*y*) denotes the fuzzy output value due to the first part of the fuzzy rule and a specific input *z*_0_. Using the same line of reasoning, we obtain the fuzzy responses due to the other antecedents and consequences along with the input *z*_0_, which are as follows:

(5) $$\begin{array}{c} \begin{array}{l} Q_{2}\left(y\right)=\min\left\{\mu_{gray}\left(z_{0}\right),\mu_{2}\left(z_{0},y\right)\right\},\\ Q_{3}\left(y\right)=\min\left\{\mu_{bright}\left(z_{0}\right),\mu_{3}\left(z_{0},y\right)\right\}. \end{array} \end{array}$$

These equations represent the result of the implication process. We should keep in mind that each of these responses is given in a fuzzy set, even though the input is a scalar value.

The application of aggregation method to the above fuzzy sets to obtain the overall response generated by the rule is carried out, and this is achieved through an *OR* operation as suggested by the proposed fuzzy rule base, that is,

(6) $$\begin{array}{c} Q\left(y\right)=\underset{r}{\max}\left\{\underset{s}{\min}\left\{\mu_{s}\left(z_{0}\right),\mu_{r}\left(z_{0},y\right)\right\}\right\}, \end{array}$$

*r* = {1, 2, 3} being the number of fuzzy outputs and *s* = {dark, gray, bright}. We can see that the overall response is the union of the three individual fuzzy sets. And this is the complete output corresponding to a specific input. But we are still dealing with a fuzzy set, so the last step is to obtain a crisp output *y*_0_. This is achieved through defuzzifying the final output fuzzy set *Q* obtained above; that is, obtaining a crisp, scalar output brought in through computing the center of gravity of *Q*, that is,

(7) $$\begin{array}{c} y_{0}=\frac{\sum_{y=1}^{p}yQ\left(y\right)}{\sum_{y=1}^{p}Q\left(y\right)}, \end{array}$$

*y*_0_ being the crisp output, *p* being the number of all possible values that *Q*(*y*) in (6) may have, and *y* being a scalar value representing the intensity levels of the pixels in output fuzzy sets.

In this way, we are able to achieve dynamic range expansion of the contrast in an efficient way using simple computation operations to conditionate the image to the future processes.

#### 2.1.1. Experimental Results of the Contrast Enhancement

Our contrast enhancement experiment was carried out using different images from different MRIs, and the proposed fuzzy set-based technique proved to be the most effective and this can be proven by the histogram shapes obtained in Figure 1. By examining the outcome of our method and comparing it to that of other classical methods, a clear difference is noted.

**Figure 3:**
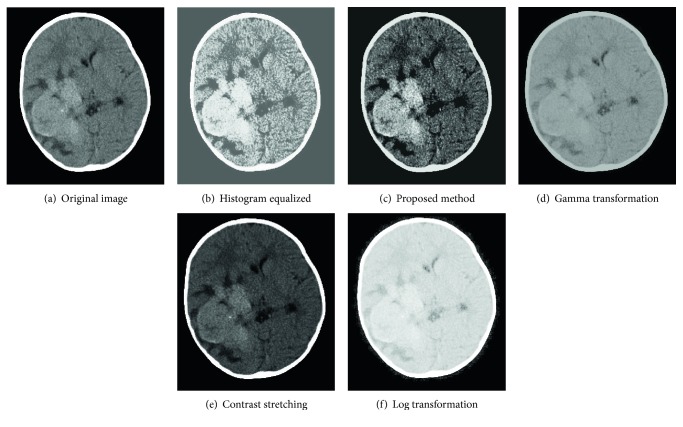
Comparison of different enhancement methods.

Let us now examine our results.  Figure 3(a) shows an image whose intensities span a narrow range of the gray scale, as the histogram in Figure 1(a) reveals. The next result is an image with low contrast; Figure 3(b) is the result of using the histogram equalization to increase image contrast. As shown by the histogram in Figure 1(b), the gray scale was a bit spread out, but shifted to the right, and it completely lost the shape of the histogram of the original image. The result was an image with an overexposed appearance. We can see that it would be quite difficult to distinguish the healthy from the unhealthy tissues due to some gray details that are lost. As we can see in Figure 3(c), the result of the proposed method is an image having increased contrast and a rich tonality. The reason for this improvement can be explained by examining the histogram in Figure 1(c). Unlike the histograms produced by other techniques, this histogram has kept the same basic characteristics of the histogram of the original image [8]. And the spread of the gray scale occurred in all directions and this applies to all MR images tested. As for the gamma and Log transformations, we can see that a lot of details were lost, as proved by their histograms. Contrast stretching was a bit more productive, but its histogram lacked stretching and this resulted in a negligible enhancement.

### 2.2. Lesion Detection Process through Segmentation

Generally, the principal goal of segmentation is to partition an image into regions (also called classes or subsets) that are homogeneous with respect to one or more characteristics or features [11]. This is very important in medical imaging, since it allows feature extraction, image measurement, and display. Most importantly, it permits the classification of image pixels into anatomical or pathological regions such as lesion and tissue deformities, amongst others.

#### 2.2.1. Unified Gravitational Fuzzy Clustering (UGFC)

Some ground-breaking MR image segmentation approaches have been developed by the most prominent researchers and run on modern processors. These include the work by Prastawa et al. [12] who, with their automatic, multimodal, atlas-based method, have reported 86.7% average overlap on a small dataset of three patients with an average 1.5 h processing time. In a recent study, Hamamci et al. [13] reported an 80%–90% overlap performance, with their method named “Tumor-cut,” which is based on the cellular automata (CA) algorithm. A serious drawback with this method, however, is that it requires diameter drawing initialization, which raises its computation time to about 16 minutes and prevents it from being fully automatic. Menze et al. [14] reported 60% average overlap on 25 glioma patients with their method which is based on the discriminative random decision forests framework. Gooya et al. [15] reported 74.5% average overlap on 15 glioma patients with about 6–14 hours of processing time, with their methods which are based on EM algorithm. Geremia et al. [16] adopted a discriminative random decision forest framework that gave good results in high-quality MRI with low noise level and high resolution. Another interesting work was by Liu et al. [17] who used the classical fuzzy clustering-based method and reported a 95.6% average overlap on a well-performing five-patient dataset of FLAIR images. The latter method needed intensive user interaction and correction at least 8.4 minutes per patient. Most recently, Shen et al. [18] extended the Fuzzy C-Means (FCM) approach by introducing an additional term describing the distance between the fuzzy membership and the prior tissue probability maps; they used a simulated image dataset, and an overlap varying from 34% to 79% was reported, depending on the signal reduction.

In this paper, we are proposing a novel segmentation method based on a combined hard and fuzzy clustering framework. This method adopts the Newtonian gravity concept from a clustering perspective in order to hard-cluster image pixels that otherwise could unnecessarily be assigned membership to clusters that they categorically do not belong to. However, this method fuzzy-clusters those pixels that are located in controversial areas in order to optimize the partial volume effect handling. The result is a well-defined region of interest (ROI) that is made up of unhealthy tissues on the MR image under consideration.

Generally, Newton's gravity law can be formulated as follows:

(8) $$\begin{array}{c} F=l\frac{m_{1}\cdot m_{2}}{d^{2}}. \end{array}$$

*F* denotes the gravity force between object 1 with mass *m*_1_ and object 2 whose mass is *m*_2_, *d* represents the distance between the two objects, and *l* is a coefficient that takes the place of Newton's constant; we set *l* = 2 for computational convenience. To apply this law effectively, we make the following assumptions:

- The quality of each pixel is 1.
- *m*_*i*_^(*t*)^ pixels have been clustered into cluster *i* at time *t*.
- Each pixel belonging to a cluster has the same potential, that is, equal preference.

All pixels in a cluster flow into an object whose mass is equal to the number of these pixels. Based on the above assumptions, the gravity force *F*^(*t*)^ between *k*th pixel and *i*th cluster will be

(9) $$\begin{array}{c} F^{\left(t\right)}=l\frac{1\times m_{i}^{\left(t\right)}}{d^{2}}=\frac{2m_{i}^{t}}{d^{2}}=\frac{2m_{i}^{t}}{{\left(x_{k}-v_{i}\right)}^{2}}. \end{array}$$

*x*_*k*_ is the *k*th pixel and *v*_*i*_ is the *i*th cluster center.

Now, we will define the gravitational clustering objective function (*J*_GC_) that should be minimized:

(10) $$\begin{array}{c} J_{GC}=\overset{c}{\underset{i=1}{\sum }}\sum_{x_{k}\in S_{i}}d^{2}\left[x_{k},v_{i}\right], \end{array}$$

where *S*_*i*_ denotes the set of pixels clustered to *i*th cluster and *c* stands for the total number of clusters in consideration. Now, as stated in [19], the standard Fuzzy *C*-Means objective function (*J*_CM_) is defined as

(11) $$\begin{array}{c} J_{CM}\overset{c}{\underset{i=1}{\sum }}\overset{N}{\underset{j=1}{\sum }}{\left(\mu_{ij}\right)}^{w}{\left(d_{ij}\right)}^{2}. \end{array}$$

**Figure 4:**
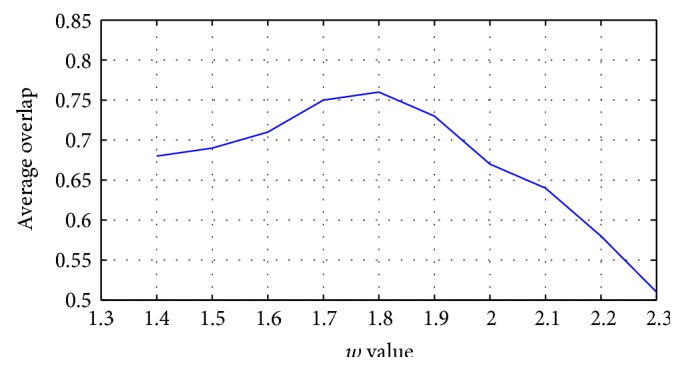
The effect of fuzzifier *w* on the segmentation performance.

*N* is the number of pixels, and *μ*_*ij*_ is the membership of *j*th pixel in the *i*th cluster and is defined as

(12) $$\begin{array}{c} \mu_{ij}^{\left(t+1\right)}={\left[\overset{c}{\underset{k=1}{\sum }}{\left(\frac{d_{ij}^{\left(t\right)}}{d_{kj}^{\left(t\right)}}\right)}^{2/\left(w-1\right)}\right]}^{-1},with\overset{c}{\underset{i=1}{\sum }}\mu_{ij}=1, \end{array}$$

where *t* is the iteration number, *w* being the fuzzifier, and *w* ∈ (1.4, 2.6), as was recently proven by Ozkan and Turksen [20] in their study based on Taylor expansion analysis of the membership value calculation function. In our experiment, *w* was chosen to be 1.7 and 1.8 since these two numbers gave the best clustering. For our three datasets, the effect of this parameter on the segmentation performance in terms of Dice overlap measure is plotted in Figure 4. Now, we will define an integrated gravitational fuzzy clustering objective function (*J*_GFC_):

(13) $$\begin{array}{c} J_{GFC}=J_{GC}\cdot J_{CM}. \end{array}$$

Taking the first derivatives of *J*_GFC_ with respect to *v*_*i*_ and setting them to zero results in the following linear systems:

(14) $$\begin{array}{c} \frac{\partial J_{GFC}}{\partial v_{i}}=J_{CM}\cdot \frac{\partial J_{GC}}{\partial v_{i}}+J_{GC}\cdot \frac{\partial J_{CM}}{\partial v_{i}}=0. \end{array}$$

**Figure 5:**
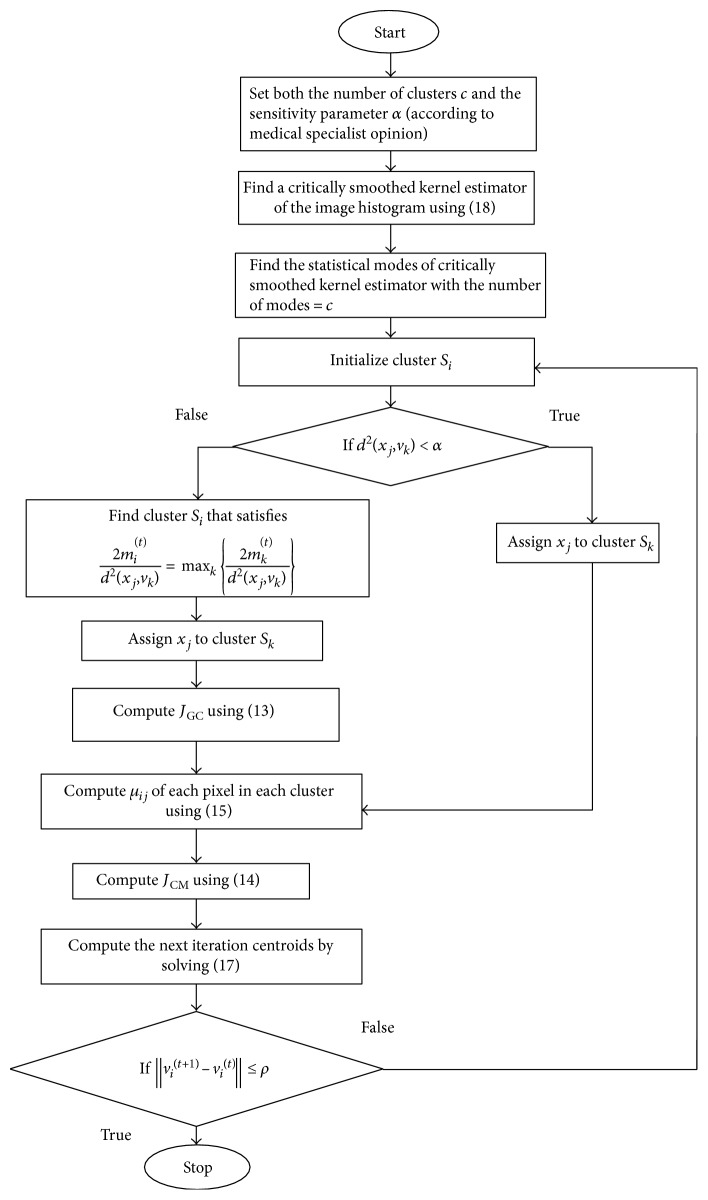
UGFC algorithm flow diagram.

Based on these equations, the integrated gravitational fuzzy clustering algorithm is structured as indicated in Figure 5. *ρ* being the convergence criterion, in our experiments, it was set to 0.01. It should be noted that the kernel estimator of the image histogram which was used in defining initial centroids, that is, *v*^(0)^ = {*v*_1_^(0)^, *v*_2_^(0)^,…,*v*_*C*_^(0)^}^*t*^, at *t* = 0, is defined as

(15) $$\begin{array}{c} \hat{p}\left(x\right)=\frac{1}{Nh}\overset{N}{\underset{j=1}{\sum }}\psi \left(\frac{x-x_{j}}{h}\right), \end{array}$$

where *ψ* is the kernel function—a Gaussian function with zero mean and variance equal to 1; *N* is the total number of pixels; *x* represents the intensity levels; and *h* ∈ (0, 50) is the bandwidth. Moreover, the critical *h*(*h*_crit_) is defined as the minimum value of *h* such that $\hat{p}$ has *c* modes that correspond to the number of clusters.

#### 2.2.2. Level Set Evolution on a Constructed Region of Interest

The contour definition of the constructed region of interest (ROI) which is based on the level set evolution without reinitialization constitutes an important part of our proposed method. This is because the clinical expert segmentation, particularly in neuroimaging, mainly outlines the edges of the ROI using manual contouring, for either surgery planning, radiotherapy, or treatment response analysis [21, 22]. A variational formulation was proposed which consists of an internal energy term that penalizes the deviation of the level set function from a signed distance function, along with an external energy term that drives the motion of the zero-level set toward the ROI boundaries set by the *GFC* algorithm. This approach had the following advantages over the classical level set formulation [23, 24]:

- The contours represented by the level set function could break or merge naturally during the evolution, and the topological changes are thus automatically handled.
- The level set function always remains a function on a fixed grid, which permits efficient numerical schemes (a finite difference scheme in our case).
- Due to the internal energy, the level set function (LSF) is naturally and automatically kept as an approximate signed distance function during the evolution. Consequently, reinitialization is avoided.

Traditionally, if *Ω_ϕ_* is a subset of the Euclidean space with a smooth boundary, then the signed distance function (SDF) of this subset is differentiable practically everywhere, and its gradient satisfies the Eikonal equation [25], that is,

(16) $$\begin{array}{c} \;|\;\nabla \phi \;|\;=1. \end{array}$$

So, any function *ϕ* satisfying this property is a SDF plus a constant. Now, we propose the following integral:

(17) $$\begin{array}{c} P\left(\phi \right)=\frac{1}{2}\int_{\Omega }{\left(\left|\nabla \phi \right|-1\right)}^{2}dxdy, \end{array}$$

as a metric that defines how close *ϕ* is to a SDF in Ω ⊂ *R*^2^ and we call it “internal energy.” Having *P*(*ϕ*) on our disposal, we then propose the following variational formulation:

(18) $$\begin{array}{c} \varepsilon \left(\phi \right)=\beta P\left(\phi \right)+\varepsilon_{m}\left(\phi \right), \end{array}$$

*β* ∈ [0.04, 0.1] is a parameter controlling the effect of penalizing the deviation of *ϕ* from a SDF, and *ε*_*m*_(*ϕ*) is the external energy that drives the motion of zero level curve of *ϕ* and depends upon the image data.

Now, we consider ∂*ε*/∂*ϕ* as being the Gateaux derivative of *ε* [26], and the evolution equation,

(19) $$\begin{array}{c} \frac{\partial \phi }{\partial t}=-\frac{\partial \varepsilon }{\partial \phi }, \end{array}$$

becomes the gradient flow that minimizes *ε*. Let *I* be the image generated by the GFC algorithm and *g* the edge indicator function that regularizes *ε*(*ϕ*) in order to stop level set evolution near the optimal solution [27]. The latter is defined as

(20) $$\begin{array}{c} g=\frac{1}{1+{\left|\nabla G_{\sigma }*I\right|}^{2}}, \end{array}$$

where *G*_*σ*_ is the Gaussian kernel with standard deviation *σ*. We then define external energy for *ϕ*(*x*, *y*) as

(21) $$\begin{array}{c} \varepsilon_{g,\lambda ,v}\left(\phi \right)=\lambda L_{g}\left(\phi \right)+{vA}_{g}\left(\phi \right), \end{array}$$

with

(22) $$\begin{array}{c} L_{g}\left(\phi \right)=\int_{\Omega }g\delta \left(\phi \right)\;|\;\nabla \phi \;|\;dxdy, \end{array}$$

and

(23) $$\begin{array}{c} A_{g}\left(\phi \right)=\int_{\Omega }gH\left(-\phi \right)dxdy, \end{array}$$

where *λ* ∈ [2,6] and *v* ∈ [1, 3.5] are constants, *δ* is the univariate Dirac function, and *H* is the Heaviside function. So, the total energy becomes

(24) $$\begin{array}{c} \varepsilon \left(\phi \right)=\beta P\left(\phi \right)+\varepsilon_{g,\lambda ,v}\left(\phi \right). \end{array}$$

Now, to understand the geometrical meaning of *L*_*g*_(*ϕ*), suppose that the zero level set of *ϕ* is represented by a differentiable parameterized curve *K*(*τ*) with *τ* ∈ [0, 1]. Then, according to Vemuri and Chen [23], *L*_*g*_(*ϕ*) computes the length of the zero level curve of *ϕ* in the conformal metric *ds* = *g*(*K*(*τ*))∣*K*^'^(*τ*)∣*dτ*. Note that when function *g* is a constant one, *A*_*g*_(*ϕ*) becomes the area of the region *Ω*_*ϕ*_ = {(*x*, *y*) | *ϕ*(*x*, *y*) < 0} [28]. *v* of *A*_*g*_ should be positive if the initial contours are placed outside the ROI and negative when they are placed inside, to speed up the contraction or the expansion, respectively. By calculus of variations, the Gateaux derivative of *ε*(*ϕ*) can be written as [26]

(25) $$\begin{array}{c} \frac{\partial \varepsilon }{\partial \phi }=-\beta \left[\Delta \phi -\mathrm{div}\left(\frac{\nabla \phi }{\left|\nabla \phi \right|}\right)\right]-\lambda \delta \left(\phi \right)\mathrm{div}\left(g\frac{\nabla \phi }{\left|\nabla \phi \right|}\right)-vg\delta \left(\phi \right). \end{array}$$

Therefore, the function *ϕ* that minimizes this function satisfies the Euler-Lagrange equation:

(26) $$\begin{array}{c} \frac{\partial \varepsilon }{\partial \phi }=0. \end{array}$$

So, the gradient flow that minimizes *ε* is

(27) $$\begin{array}{c} \frac{\partial \phi }{\partial t}=-\beta \left[\Delta \phi -\mathrm{div}\left(\frac{\nabla \phi }{\left|\nabla \phi \right|}\right)\right]+\lambda \delta \left(\phi \right)\mathrm{div}\left(g\frac{\nabla \phi }{\left|\nabla \phi \right|}\right)-vg\delta \left(\phi \right), \end{array}$$

which is the evolution equation of the level set function in the proposed algorithm. To explain the effect of the first term in the right-hand side of equation above, that is, *βP*(*ϕ*) which is the internal energy, we notice that the gradient flow:

(28) $$\begin{array}{c} \Delta \phi -\mathrm{div}\left(\frac{\nabla \phi }{\left|\nabla \phi \right|}\right)=\mathrm{div}\left[\left(1-\frac{1}{\left|\nabla \phi \right|}\right)\nabla \phi \right], \end{array}$$

has the factor (1 − (1/|∇*ϕ*|)) as its diffusion rate. If |∇*ϕ*| > 1, the diffusion rate is positive and the effect of this term is the usual diffusion, that is, making *ϕ* more even and therefore reduce the gradient |∇*ϕ*|. If |∇*ϕ*| > 1, then the term has effect of reverse diffusion and therefore increases the gradient [29].

## 3. Evaluation and Experimental Results

Quantitative and qualitative validation studies of the developed method were conducted over three different datasets. These sets comprise multimodal MR images (T1, T1Gd, T2, and FLAIR) of 80 low-grade and high-grade gliomas from synthetic and real patient cases, with 1 mm of isotropic resolution. These datasets were obtained from the following:

- University of Utah database [30]: Synthetic brain tumor datasets were used in the first part of validation. This data simulates contrast-enhanced T1-weighted MR images with synthetically generated tumors. The tumor probability maps and levels of intensity nonuniformity (bias field) are also available. This dataset is included in the performance evaluations since the ground truth segmentation is readily available.
- Real MR images with ground truth from Kitware database (KIT) [31]: KIT offers both synthetic and real brain images with manually guided expert segmentation results.
- Brain tumor datasets obtained from our INNN: A large dataset of brain tumor/lesion patients, who received treatment from the Instituto Nacional de Neurología y Neurocirugía (INNN), Mexico, was utilized in the second set of experiments. As a ground truth for our segmentation phase, we used the tumor contours outlined manually by a radio-oncology specialist in the same hospital. These images were both T1-weighted and FLAIR modalities, and they provided an effective way to suppress cerebrospinal fluid (CSF) to bring out periventricular hyperintense lesions. It is worth mentioning that a 1.5 T MRI scanner located at INNN was used to generate the images.

To evaluate the segmentation quantitatively, we used the Jaccard coefficient (*R*) and the Dice overlap (*D*) [32], for similarity measurement and sensitivity (Se) and specificity (Sp) for success and error rate measurements [33]. These measures can be expressed as

(29) $$\begin{array}{c} R\left(T,S\right)=\frac{\left|T\cap S\right|}{\left|T\cup S\right|}=\frac{\left|TP\right|}{\left|TP\right|+\left|FP\right|+\left|FN\right|}, \end{array}$$

(30) $$\begin{array}{c} D\left(T,S\right)=\frac{2\left|T\cap S\right|}{\left|T\right|+\left|S\right|}=\frac{2\left|TP\right|}{\left|TP\right|+\left|FP\right|+\left|FN\right|}, \end{array}$$

(31) $$\begin{array}{c} Se=\frac{\left|TP\right|}{\left|T\right|}=\frac{\left|TP\right|}{\left|TP\right|+\left|FN\right|}, \end{array}$$

(32) $$\begin{array}{c} Sp=\frac{\left|TN\right|}{\left|\bar{T}\right|}=\frac{\left|TN\right|}{\left|TN\right|+\left|FP\right|}, \end{array}$$

where *T* = ground truth, *S* = pixels labelled by the algorithm, TP = true positive, FP = false positive, and FN = false negative. As was previously mentioned, experiments were conducted on both real and synthetic images.

**Figure 6:**
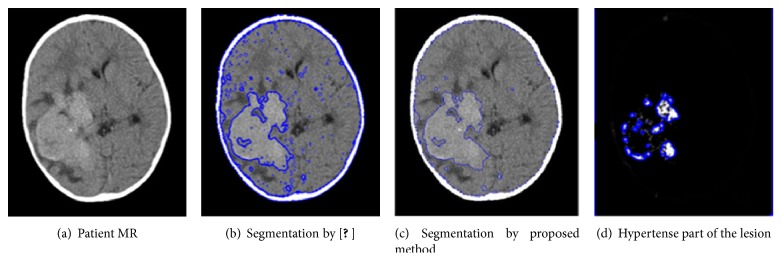
Lesion contour calculation.

Figure 6 shows the same challenging patient's MR image demonstrated in Figure 2, whose contrast is very poor and has very small intensity shift along the lesion edges, making the detection more complex. This can be witnessed in Figure 2(b), where after the enhancement, detection was carried out by a robust system described in [33] and ended up being misleading due to the intensity inhomogeneity present in the image. However, by applying the proposed method, the lesion was detected with greater accuracy, as seen in Figure 6(c). This improvement owes a lot to the hard-fuzzy clustering, which reflects the gravity concept. Generally, in classical fuzzy clustering, all pixels in an image are assigned a membership to every structure, regardless of the magnitude of the corresponding membership. This makes it easier, as we discovered in our experiment, for some pixels to be misclassified especially when they fall into the intensity range of the structure. For this reason, when considering the presence of some random intensity-inhomogeneities that are spread across the image and physically linked not to the tissue problem but rather to the radiofrequency MR signal, one needs not only to consider the intensity level of the pixel but also its spatial location in order to guarantee a proper clustering. Spatial location of pixels is of great importance because it helps us carry out a gravitation-based clustering in areas where fuzzy clustering is not suitable. For instance, pixels at the center of WM/GM tissues could unnecessarily be assigned membership to other tissues that they do not in fact belong to; this is why it is preferable that they are hard-clustered. Applying the gravitation concept, these same pixels will attract all the other pixels located not far from this neighborhood as long as *d*^2^ is small enough and *m*_*i*_^(*t*)^ is big enough to outweigh the attraction by other clusters. Moreover, they will be joined together to form a stronger cluster as was explained in the algorithm. On the other hand, pixels located in the intersection zone of tissues, which have an intensity range that could make them fit in any of these tissues, would do better if fuzzy-clustered. This would eliminate every sort of uncertainty, take care of partial volume effect, and reach optimal state more promptly. The advantages of this gravitational fuzzy clustering phenomenon can be explicitly witnessed in Figure 6(c), where all the pixels wrongly assigned to the lesion in Figure 6(b) just because their intensity range matched that of the lesion were discounted in Figure 6(c) not because of their intensity but because of their spatial location.

**Figure 7:**
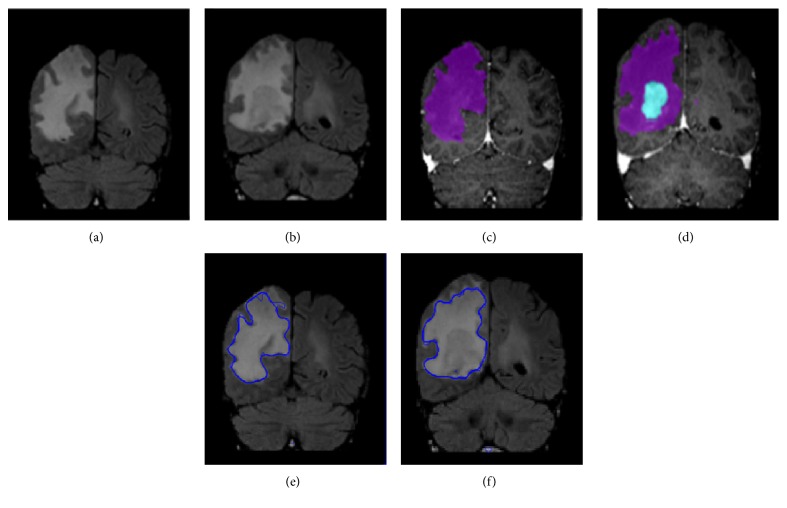
FLAIR images (a, d), T1-Gad ground truth (b, e), and T1-Gad proposed method (c, f). KIT001 (a, b, c) and KIT002 (d, e, f).

The same scenario can be witnessed in Figure 7 where each of the two FLAIR images on the top row carries a lesion on the left frontal lobe, but there is also a seemingly detectable lesion on the right frontal lobe. However, the algorithm disregarded it, despite its convincing intensity range, which is in accordance with the ground truth in the second row.

**Figure 8:**
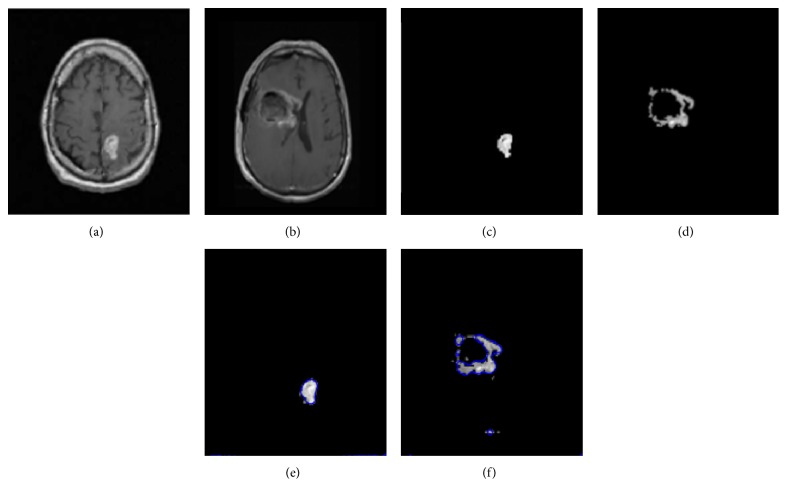
T1-image (a, b), ground truth (c, d), and proposed method (e, f). INNN1 (a, c, e) and INNN2 (b, d, f).

**Figure 9:**
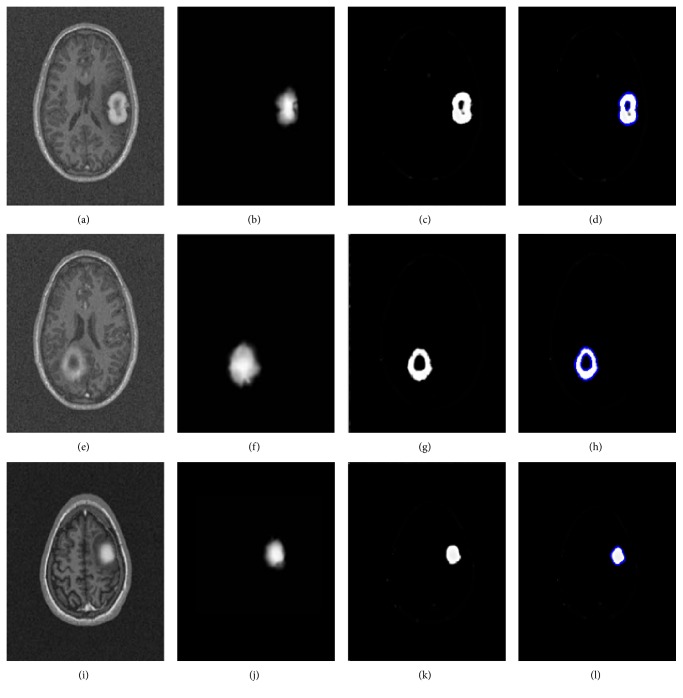
MR images of the synthetic dataset. syn001 (a, b, c, d), syn002 (e, f, g, h), and syn003 (i, j, k, l). Original image (a, e, i), ground truth (b, f, j), GFC-result (c, g, k), and final result (d, h, l).

**Figure 10:**
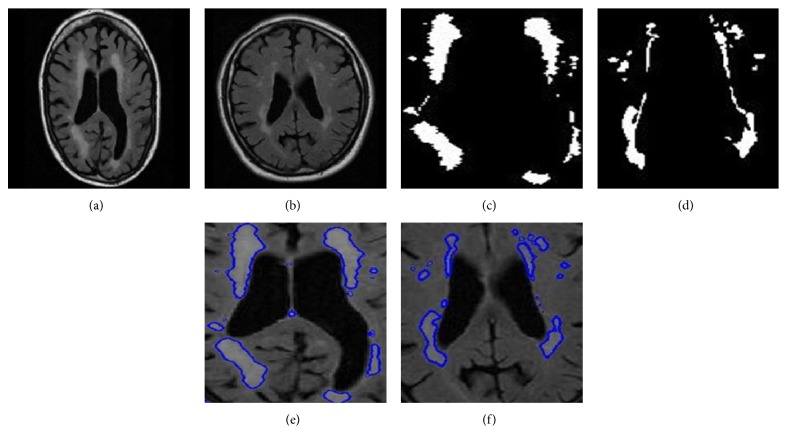
Multiple sclerosis cases. INNN17 (a, c, e) and INNN21 (b, d, f). Original image (a, b), ground truth (c, d), and proposed method (e, f).

**Figure 11:**
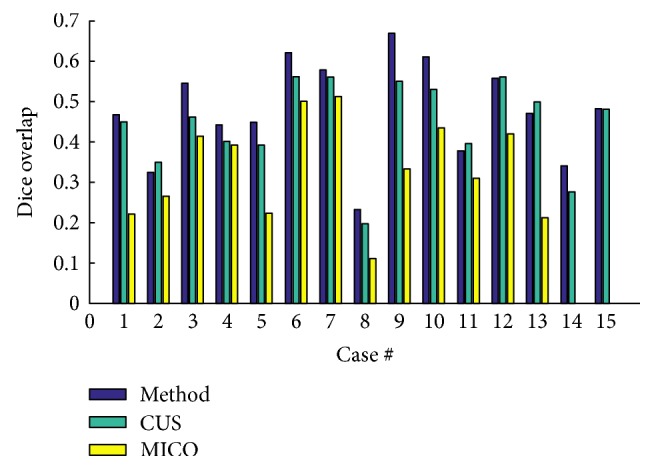
Comparison of the proposed method against CUS and MICO methods as carried out on the synthetic dataset.

**Figure 12:**
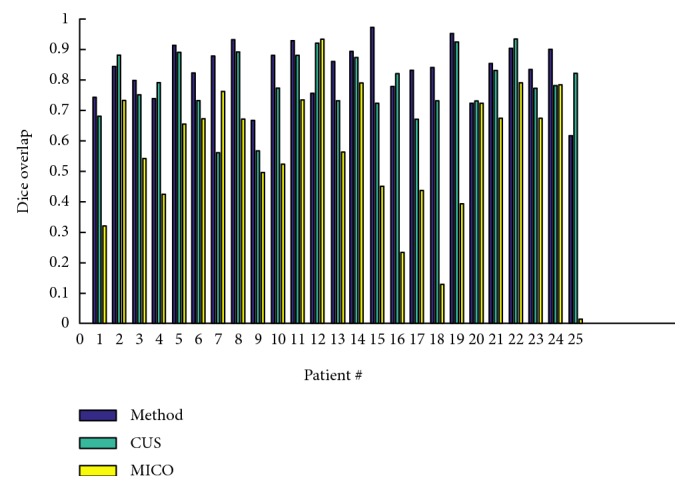
Comparison of the proposed method against CUS and MICO methods as carried out on the Kitware dataset.

**Figure 13:**
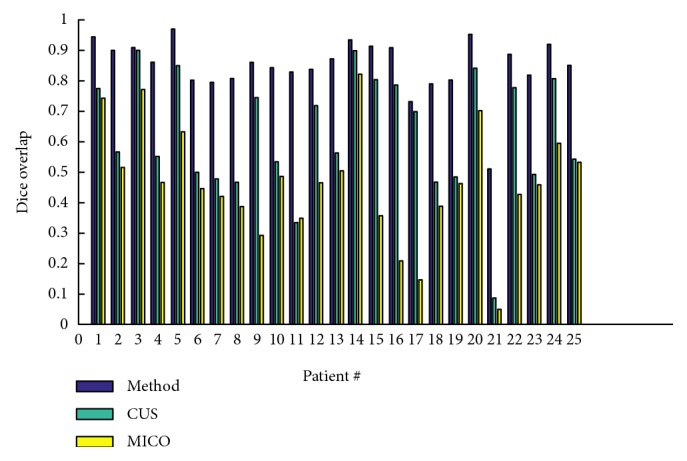
Comparison of the proposed method against CUS and MICO methods as carried out on INNN dataset.

Figure 8 portrays the segmentation results for the two high-grade gliomas, and we can see that the *UGFC* results in the third row perfectly match the ground truth in the second row. As mentioned before, evaluation studies were carried out using (29), (30), (31), and (32). Furthermore, performance measures, that is, Dice overlap, Jaccard, sensitivity, and specificity between the ground truth segmentation and the result of the algorithm are reported in Tables 1, 2, and 3 for both synthetic and clinical datasets. Good results on real MR images were obtained as can be seen in Tables 2 and 3, which demonstrate the performance on Kitware datasets and Instituto Nacional de Neurología y Neurocirugía datasets. However, this was not the case on synthetic images.  Figure 9 shows some synthetic MR images from the University of Utah database where the detection was successful; however, as demonstrated in Table 1, a poor overlap performance was obtained due to a misleading ground truth. Nevertheless, based on visual inspection, one can see that the detection was good enough.  Figure 10 shows more challenging cases of patients with multiple sclerosis (INNN17, INNN21), where the intensity spectrum of the lesion almost matches that of the healthy tissues. Moreover, the lesions present a high degree of discontinuity which was a big challenge to this algorithm, thereby resulting in a very poor performance as can be seen in Table 1 where INNN17 has a 0.732 overlap and INNN21 has a 0.511 performance. We also compared the proposed method against the Automated Lesion Detection on MRI Scans Using Combined Unsupervised and Supervised Methods by Guo et al. [34] and Multiplicative Intrinsic Component Optimization (MICO) [35] method by Li et al., and their results are reported in Figures 11, 12, and 13.

**Figure 14:**
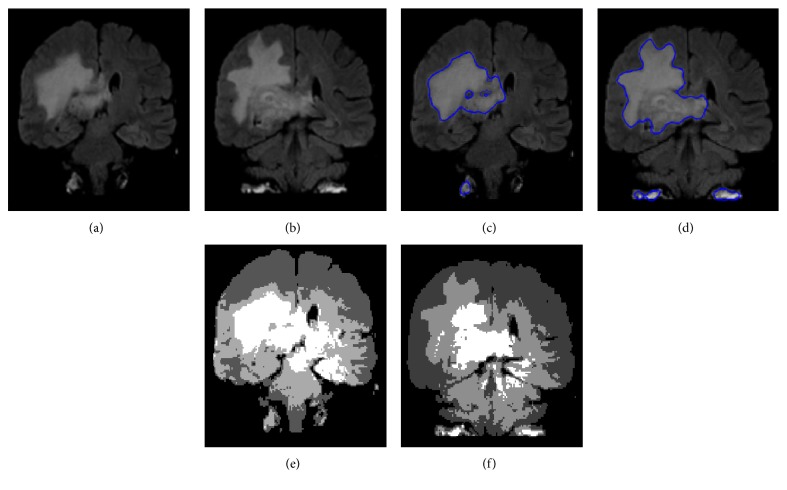
Comparison between proposed method (c, d) and Multiplicative Intrinsic Component Optimization (MICO) (e, f).

Finally, Figure 14 shows two KIT images processed by the proposed method and the Multiplicative Intrinsic Component Optimization (MICO), and it can be seen that a visual inspection witnesses a much more precise detection by our method than the MICO method. We should emphasize that the intensity inhomogeneity presented in Figure 14 did confuse the MICO method, which went even beyond the lesion contour. Yet, our method got it right.

## 4. Discussion and Conclusions

We presented a brain lesion detection algorithm along with validation studies over a synthetic lesion dataset and two real datasets: one from Kitware Repository and another from a clinical database of tumors that underwent radiosurgery planning at Instituto Nacional de Neurología y Neurocirugía de México (INNN). The performance over these datasets of highly heterogeneous tissue content demonstrated an average overlap of 0.83 and 0.85. However, a poor performance of 0.48 was registered in synthetic MR images from the Utah database, mostly due to the poor ground truth presented. In all cases, the proposed algorithm provided superior quality segmentation when compared to the benchmark algorithms. On an average, the proposed automated algorithm takes about 8 seconds (measured using MATLAB R2007b on a 3.00 GHz, dual processor) in comparison to 21 and 12 seconds taken by the MICO and CUS respectively. Strengths of the proposed method include its automatic nature, its efficiency in terms of computation time, and its robustness with respect to different and heterogeneous lesion types. Another important aspect to consider is that the algorithm can work on different MR modalities.

## Figures and Tables

**Table 1:** Performance criteria of the proposed method applied on synthetic images from Utah database: Dice, Jaccard, sensitivity, and specificity score.

ID	Dice	Jaccard	Sensitivity	Specificity
Syn001	0.467	0.341	0.654	0.993
Syn002	0.324	0.284	0.525	0.991
Syn003	0.545	0.439	0.713	0.997
Syn004	0.442	0.322	0.611	0.991
Syn005	0.449	0.328	0.613	0.994
Syn006	0.621	0.564	0.784	0.998
Syn007	0.578	0.458	0.733	1
Syn008	0.233	0.169	0.499	0.989
Syn009	0.669	0.587	0.795	0.999
Syn010	0.611	0.536	0.743	0.997
Syn011	0.378	0.381	0.416	0.987
Syn012	0.558	0.431	0.701	0.992
Syn013	0.471	0.351	0.662	0.995
Syn014	0.342	0.299	0.546	0.994
Syn015	0.482	0.359	0.666	1
Mean	0.478	0.390	0.644	0.994
Standard deviation	0.122	0.114	0.109	0.004

**Table 2:** Performance criteria of the proposed method applied on low-grade/high-grade images from Kitware: Dice, Jaccard, sensitivity, and specificity score.

ID	Dice	Jaccard	Sensitivity	Specificity
KIT001	0.742	0.611	0.793	0.999
KIT002	0.843	0.703	0.852	1
KIT003	0.798	0.731	0.884	1
KIT004	0.738	0.601	0.785	0.993
KIT005	0.913	0.852	0.908	1
KIT006	0.822	0.765	0.925	1
KIT007	0.877	0.797	0.932	1
KIT008	0.932	0.868	0.958	1
KIT009	0.668	0.526	0.790	0.994
KIT010	0.880	0.798	0.912	1
KIT011	0.929	0.862	0.947	1
KIT012	0.757	0.630	0.818	0.999
KIT013	0.862	0.756	0.924	1
KIT014	0.893	0.828	0.939	1
KIT015	0.971	0.890	0.975	1
KIT016	0.778	0.661	0.792	1
KIT017	0.832	0.758	0.922	1
KIT018	0.840	0.748	0.852	1
KIT019	0.952	0.898	0.968	1
KIT020	0.722	0.665	0.825	0.996
KIT021	0.852	0.795	0.925	1
KIT022	0.902	0.838	0.928	1
KIT023	0.834	0.769	0.923	1
KIT024	0.898	0.841	0.944	1
KIT025	0.618	0.478	0.750	0.992
Mean	0.834	0.747	0.887	0.999
Standard deviation	0.088	0.112	0.067	0.002

**Table 3:** Performance criteria of the proposed method applied on high-grade/low-grade tumor images from INNN: Dice, Jaccard, sensitivity, and specificity score.

ID	Dice	Jaccard	Sensitivity	Specificity
INNN001	0.942	0.851	0.943	1
INNN002	0.899	0.785	0.926	1
INNN003	0.907	0.839	0.938	1
INNN004	0.860	0.763	0.918	1
INNN005	0.968	0.882	0.978	1
INNN006	0.801	0.701	0.880	1
INNN007	0.795	0.699	0.834	1
INNN008	0.807	0.731	0.890	1
INNN009	0.861	0.725	0.914	1
INNN010	0.842	0.711	0.906	1
INNN011	0.828	0.701	0.886	1
INNN012	0.837	0.707	0.901	1
INNN013	0.871	0.765	0.933	1
INNN014	0.933	0.831	0.940	1
INNN015	0.911	0.876	0.939	1
INNN016	0.907	0.855	0.927	1
INNN017	0.732	0.658	0.811	1
INNN018	0.790	0.684	0.839	1
INNN019	0.801	0.713	0.846	1
INNN020	0.951	0.873	0.967	1
INNN021	0.511	0.440	0.816	0.901
INNN022	0.887	0.778	0.949	1
INNN023	0.819	0.718	0.850	1
INNN024	0.918	0.877	0.942	1
INNN025	0.851	0.728	0.944	1
Mean	0.850	0.756	0.905	0.996
Standard deviation	0.091	0.097	0.048	0.019
